# Chromatin accessibility illuminates single-cell regulatory dynamics of rice root tips

**DOI:** 10.1186/s12915-022-01473-2

**Published:** 2022-12-08

**Authors:** Dan Feng, Zhe Liang, Yifan Wang, Jiaying Yao, Zan Yuan, Guihua Hu, Ruihong Qu, Shang Xie, Dongwei Li, Liwen Yang, Xinai Zhao, Yanfei Ma, Jan U. Lohmann, Xiaofeng Gu

**Affiliations:** 1 Biotechnology Research Institute, Chinese Academy of Agricultural Sciences, Beijing, 100081 China; 2 Annoroad Gene Technology, Beijing, 100176 China; 3 Centre for Organismal Studies, Heidelberg University, 69120 Heidelberg, Germany

**Keywords:** Rice, Root, Cell type, Chromatin accessibility, scATAC-seq, Developmental trajectory, Environmental stimulus

## Abstract

**Background:**

Root development and function have central roles in plant adaptation to the environment. The modification of root traits has additionally been a major driver of crop performance since the green revolution; however, the molecular underpinnings and the regulatory programmes defining root development and response to environmental stress remain largely unknown. Single-cell reconstruction of gene regulatory programmes provides an important tool to understand the cellular phenotypic variation in complex tissues and their response to endogenous and environmental stimuli. While single-cell transcriptomes of several plant organs have been elucidated, the underlying chromatin landscapes associated with cell type-specific gene expression remain largely unexplored.

**Results:**

To comprehensively delineate chromatin accessibility during root development of an important crop, we applied single-cell ATAC-seq (scATAC-seq) to 46,758 cells from rice root tips under normal and heat stress conditions. Our data revealed cell type-specific accessibility variance across most of the major cell types and allowed us to identify sets of transcription factors which associate with accessible chromatin regions (ACRs). Using root hair differentiation as a model, we demonstrate that chromatin and gene expression dynamics during cell type differentiation correlate in pseudotime analyses. In addition to developmental trajectories, we describe chromatin responses to heat and identify cell type-specific accessibility changes to this key environmental stimulus.

**Conclusions:**

We report chromatin landscapes during rice root development at single-cell resolution. Our work provides a framework for the integrative analysis of regulatory dynamics in this important crop organ at single-cell resolution.

**Supplementary Information:**

The online version contains supplementary material available at 10.1186/s12915-022-01473-2.

## Background

Recently, single-cell RNA sequencing (scRNA-seq) has been applied to root cells of *Arabidopsis* and rice and revealed the transcriptional programmes of major cell types, as well as the heterogeneity within a given population [1–9]. However, the mechanisms underlying these programmes for distinct cell types remain largely elusive. In eukaryotes, gene expression is highly associated with open chromatin, which can be mapped by assay for transposase-accessible chromatin with high throughput sequencing (ATAC-seq) [10]. Recently, ATAC-seq has been applied to single cells of complex tissues in several multicellular eukaryotic species [11–15], including two plant species, namely *Arabidopsis* and maize [16–18]. Consistent with the important role of chromatin state for gene expression, these studies identified distinct chromatin patterns across different tissues and cell types and revealed key regulatory sequences, which in turn could be associated with transcription factors (TFs).

Similar to maize, rice is one of the most important crop plants worldwide, and we have previously profiled the single-cell transcriptomes of rice root tips from two cultivars, which revealed species-specific expression programmes, as well as more conserved pathways [7]. To elucidate the regulatory mechanisms that are responsible for the observed gene expression states, we have now investigated the landscape of chromatin accessibility at single-cell resolution. To this end, we profiled over 46,000 nuclei from rice root tips by scATAC-seq and identified almost all cell types by cell type-specific accessible chromatin regions (ACRs). We leverage these ACRs to explore the dynamics of chromatin accessibility during rice root development and in response to environmental changes.

## Results and discussion

### scATAC-seq and identification of cell type clusters

**Fig. 1:**
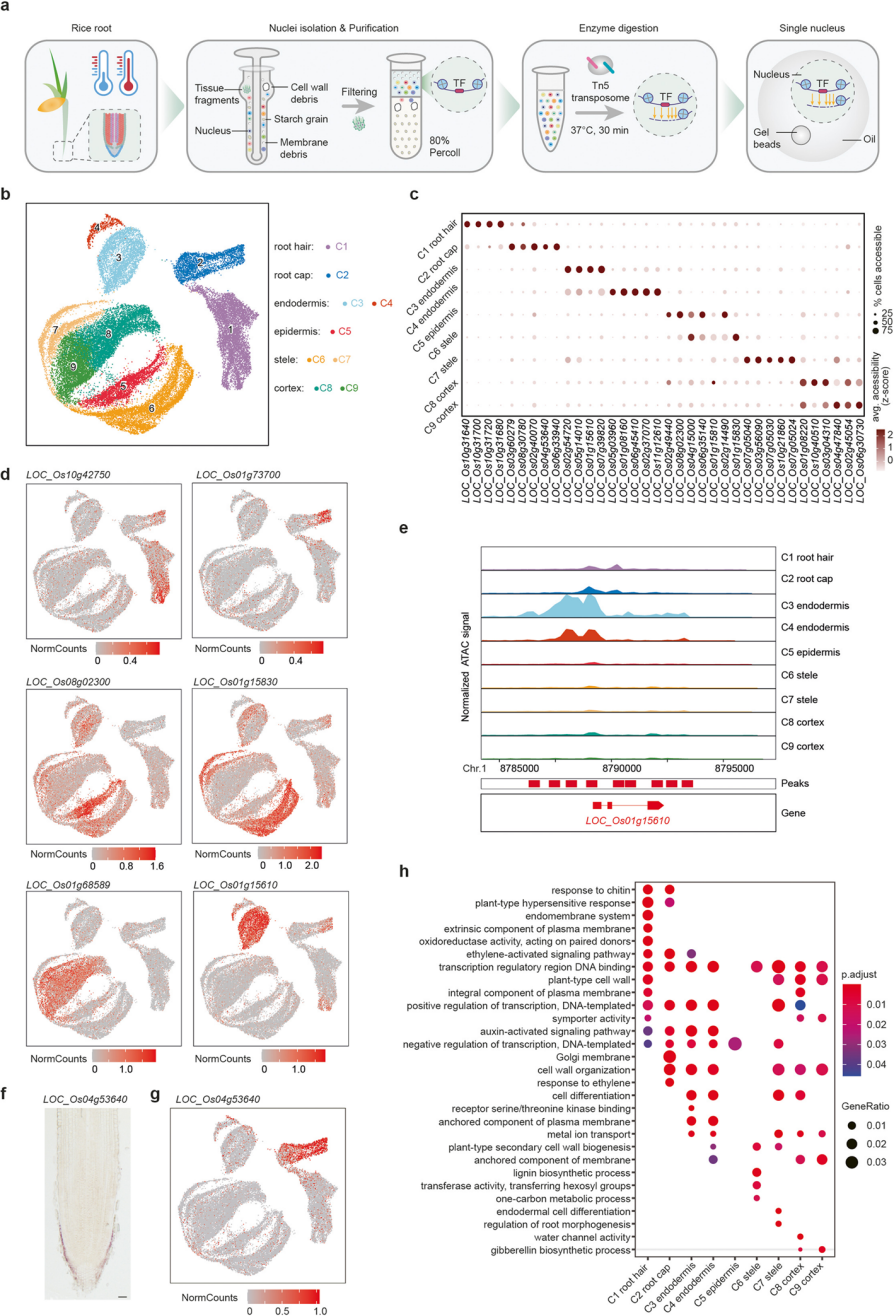
Single-cell ATAC-seq and cluster annotation of rice root tips. **a** Schematic overview of the experimental workflow. **b** UMAP visualization of nine clusters of root tip cells. Each dot indicates a single cell. Colours in the diagram of the root tip indicate the corresponding cell type. **c** Enrichment of accessibility of proximal regulatory elements for marker genes as identified by single-cell RNA-seq. Dot diameter, proportion of cluster cells with proximal regulatory elements for a given gene; colour, mean accessibility across cells in that cluster. **d** UMAP visualization of cell type-specific gene accessibility for a subset of marker genes associated with six different cell types. Each dot indicates a single cell. **e** Cluster-aggregated chromatin accessibility surrounding the gene *LOC_Os01g15610* for cells of endodermis. **f** RNA in situ hybridization of cell type accessibility marker gene *LOC_Os04g53640* for rice root cap. Scale bar, 40 μm. **g** UMAP visualization of cell type-specific gene accessibility of *LOC_Os04g53640* for rice root cap. Each dot indicates a single cell. **h** Scatter plots of GO enrichment analysis of differentially accessible genes for each cluster. Overrepresentation analysis and visualization performed using the ClusterProfiler R package. GO terms were obtained with adjusted *p*-value < 0.05 using the Benjamini–Hochberg method

We performed scATAC-seq on root tips isolated from rice *Japonica* group cultivar Nipponbare (Nip). High-quality nuclei from 120 root tips (5 mm in length of primary roots) were obtained from 3-day-old Nip seedlings using our improved nuclei isolation protocol (Additional file 1: Fig. S1). About 16,000 nuclei per replicate were incubated with Tn5 transposase in bulk. The transposed nuclei were then loaded onto a 10x Genomics microfluidic device and mixed with single-cell reaction reagents including cell barcodes. The resulting libraries were sequenced in a single pool (Fig. 1a). To exclude low-quality cells, we removed cells with less than 1000 unique nuclear fragments and a transcription start site (TSS) enrichment score of < 1.5. In addition, we removed doublets by using ArchR [19]. We found an excellent correlation between replicates, and different samples were still very similar despite being processed in different batches, suggesting that our process was robust (Additional file 1: Fig. S2a-c). For further quality control (QC), we first performed bulk ATAC-seq of independently isolated nuclei and again found a high correlation between scATAC-seq and bulk ATAC-seq (Additional file 1: Fig. S2a). Second, we compared the fragment size distribution in our samples to published data from other plants, such as maize [18], and found them to be similar (Additional file 1: Fig. S2b). Third, strong enrichment of fragments was observed within the regions that have been described as accessible chromatin (Additional file 1: Fig. S2d). Forth, the majority of scATAC peaks between replicates were overlapped (Additional file 1: Fig. S2e). Taken together, these results demonstrated that our scATAC-seq dataset is robust and of high quality. To give the community full access to the intuitive mining of our data, we have developed the Single-Cell Chromatin Accessibility of Rice root (SCAR) website, which is freely available at http://www.elabcaas.cn/scar/index.html.

Building on our quality controls, we were able to identify an average of 14,602 unique Tn5 integration sites in 25,312 nuclei, which corresponded to 13,848 ACRs covering ~ 18.6% of the rice genome (Additional file 1: Table S1). Based on unsupervised uniform manifold approximation and projection (UMAP), cells grouped into nine clusters representing distinct chromatin profiles (Fig. 1b). Following published strategies [15, 18], we annotated clusters based on specific ACRs located in the promoters of genes with cell type-specific expression. To this end, we first identified a list of genes with cluster-specific ACRs using a gene score matrix (Fig. 1c; Additional file 2: Tables S2, S3), before cross-referencing with our previously published cell type marker genes [7] (Fig. 1d). For example, the promoter of the root hair marker gene *LOC_Os10g42750* contains a cluster 1 (C1)-specific ACR, the promoter of the root cap marker gene *LOC_Os01g73700* contains a cluster 2 (C2)-specific ACR, the promoter of the endodermis marker gene *LOC_Os01g15610* contains a cluster 3 (C3)- and cluster 4 (C4)-specific ACR, the promoter of the epidermis marker gene *LOC_Os08g02300* contains a cluster 5 (C5)-specific ACR, the promoter of the stele marker gene *LOC_Os01g15830* contains a cluster 6 (C6)- and cluster 7 (C7)-specific ACR, and the promoter of the cortex marker gene *LOC_Os01g68589* contains a cluster 8 (C8)- and cluster 9 (C9)-specific ACR (Fig. 1d, e; Additional file 1: Fig. S3a). This strategy allowed us to annotate all scATAC-seq clusters and revealed that major cell types of the root were represented in our dataset. Interestingly, not all cluster-specific ACRs mapped to cell type marker genes. Consequently, we performed RNA in situ hybridization for a gene associated with differentially accessible regions and no prior evidence of cell type-specific expression. Reassuringly, the mRNA detection of *LOC_Os04g53640* in the root cap was consistent with its enriched expression in the UMAP annotated root cap cluster (Fig. 1f, g; Additional file 1: Fig. S3b). It suggests that cluster-specific ACRs have the power to detect differentially expressed genes that single-cell RNA-seq is unable to identify.

Next, we compared the scATAC-seq with our recently reported scRNA-seq data from the protoplast of rice root tips [7]. We embedded them by adopting the approach used for integrating *Arabidopsis* scRNA-seq and scATAC-seq datasets [16] and plotted all cells with cell type labels from each dataset (Additional file 1: Fig. S4a). UMAP visualization showed similar distribution patterns for matching cell types between scRNA-seq and scATAC-seq, which further supported our cluster annotation. We also observed non-overlapping cells, which are likely caused by multiple factors, including (1) significant expression effects induced by the protoplasting during scRNA-seq, absent from the scATAC-seq process, and (2) genes with open chromatin accessibility but low/no expression (Additional file 2: Table S4), and genes in regions of closed chromatin, but high expression (Additional file 2: Table S5). Overall, cell type marker genes of scATAC-seq and scRNA-seq (Additional file 2: Table S6) showed a much higher correlation between matching cell types than between different cell types (Additional file 1: Fig. S4b), suggesting that the chromatin accessibility and gene expression datasets were positively correlated, further substantiating our cluster annotation. However, we found that some cell type marker genes of scRNA-seq, including *LOC_Os08g03450* of endodermis, did not show cell type-specific chromatin accessibility. One explanation is that scATAC-seq was performed on the nuclei, while RNA in situ hybridization (scRNA-seq) was performed on the whole tissue (cell), including the cytoplasm. The regulation of RNA decay, RNA transport, RNA methylation, etc. may contribute to the differences between chromatin accessibility and gene expression. On the other hand, it suggested that further experimental validation would still be required.

To categorize the function of genes associated with cluster-specific ACRs, we performed Gene Ontology (GO) analyses and found converging terms between scATAC-seq and scRNA-seq for all cell types. For example, genes related to water transport were enriched among mRNAs of cortex cells in scRNA-seq [7], as well as genes associated with cortex-specific ACRs identified in scATAC-seq (Fig. 1h; Additional file 2: Table S7), further highlighting the close correlation between chromatin accessibility maps and cell type transcriptomes from rice root tips.

### Characterization of cell type TF motifs in rice

**Fig. 2:**
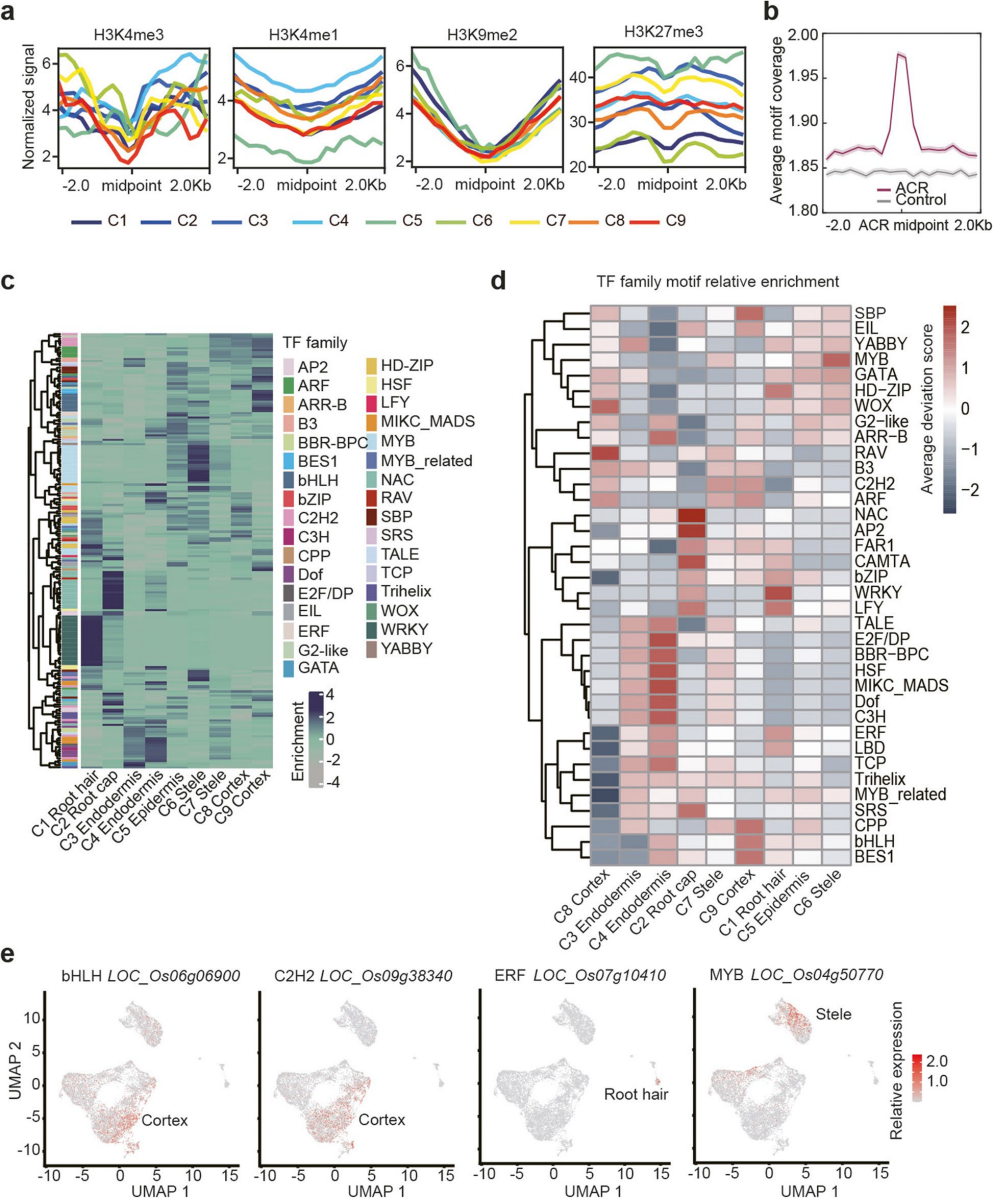
Characterization of cell type TF motifs in rice root tips. **a** Distribution of histone modifications around ACRs. Colours represent different cell types. **b** The average motif coverage across 4-kb windows centred on ACRs (*n* = 138,481) and control regions (*n* = 138,481). Shaded polygon, 95% confidence intervals. **c** Heatmap showing 264 TF motif enrichments represented by average deviation scores in the top 2000 ACRs for each cell type cluster. **d** The mean TF family motif enrichment (average deviation scores per cell type per TF family) across cell type scale by row with *z*-score. **e** Cross-referencing cluster-enriched TF binding motifs with scRNA-seq data (Liu et al. [7]); UMAP visualization of expression of cell type-specific TFs in Nip. Colour bars indicate the scaled expression level

We have previously described cell type-specific transcriptomes for root tips of two rice cultivars [7], but the regulatory mechanisms leading to these highly distinct expression programmes remained elusive. After having identified cell type-specific ACRs, we next asked which chromatin marks may be associated with these regions to gain an insight into their biochemical state. To this end, we integrated published ChIP-seq data on histone modifications with our ATAC-seq-based ACRs. Interestingly, we found that for all modifications tested, namely H3K4me1, H3K4me3, H3K27me3, and H3K9me2, the ChIP-seq signal at the middle point of ACRs represented a regional minimum. Furthermore, we found that levels of some modifications, such as H3K4me1 and H3K27me3, were different between cell types, whereas H3K9me2 appeared similar across all cell types (Fig. 2a). The underlying mechanism for this interesting pattern could be explored by future single-cell/cell type resolution histone modifications studies. These results suggested on the one hand that ACRs represent genomic regions with reduced histone methylation and on the other hand showed that these modifications show cell type-specific variations, which could contribute to chromatin accessibility and gene expression.

Since gene expression is dictated by the interplay of open chromatin with TF activity, we aimed to elucidate the regulatory networks responsible for cell type differentiation in rice roots. Our analyses were built on the notion that TF-binding elements in ACRs represent the substrate for expressed TFs, identified by scRNA-seq, since most TFs can only bind to open chromatin, which is readily identified by scATAC-seq [18]. To establish cell type TF signatures, we first annotated known TF-binding sequences across the rice genome and plotted the average motif coverage around ACRs. We found that the occurrence of potential TF-binding sites was highly enriched in ACRs relative to control and flanking regions of the rice genome (Fig. 2b), supporting the idea that ACRs represent important regulatory regions. Next, we identified highly represented TF motifs for each cell type by calculating the relative enrichment of TF binding sequences within the top 2000 differential ACRs. An average of 32 TF motif combinations were significantly enriched (Fisher exact test, FDR < 0.01) per cell type, and the largest number of motif combinations was 53, found in the C2 root cap. Our analysis revealed many cluster-enriched TF motifs (Fig. 2c, d, Additional file 1: Fig. S5). For example, WRKY family TF motifs enriched in root hair; NAC, AP2, and CAMTA family TF motifs enriched in root cap; MYB family TF motifs enriched in the stele; and C2H2 family TF motifs enriched in the cortex (Additional file 1: Fig. S6). The enrichment of TF motifs was coincident with the known regulators of cell identity, including the WRKY family in root hair development [20], NAC family TFs in root cap development [21], and DNA binding with one finger (Dof) TF in endodermis regulation [22]. Cross-referencing these findings with our scRNA-seq data, we found that some members of TFs with cell type-specific expression, such as members of bHLH, MYB, C2H2, and ERF family TFs (Fig. 2e), coincide with their enriched TF-binding motifs from the same cell type (Fig. 2d; Additional file 1: Fig. S5a), further indicating that our annotation was robust.

### Chromatin accessibility trajectory of epidermal and root hair cells

**Fig. 3:**
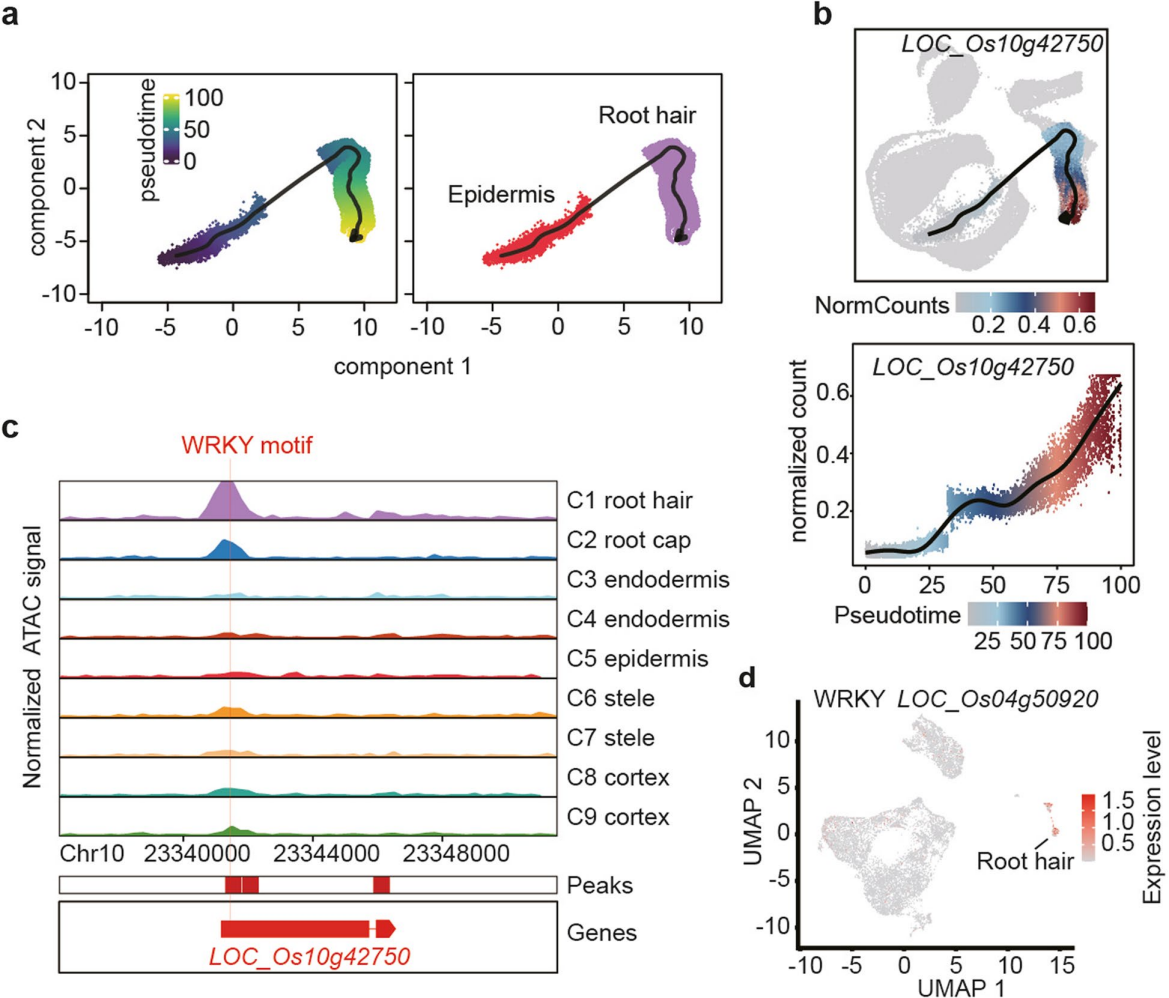
Chromatin accessibility trajectory of epidermal and root hair cells. **a** UMAP visualization of the root hair developmental trajectory depicting pseudotime (left) and cell types (right). **b** UMAP visualization of pseudotime of chromatin accessibility (top) and gene expression (bottom) for the root hair marker gene *LOC_Os10g42750*. **c** Cluster-aggregated chromatin accessibility surrounding the gene *LOC_Os10g42750* for cells of root hair. A WRKY was indicated in the ACR of *LOC_Os10g42750*. **d** UMAP visualization showing cell type-specific expression of WRKY TF *LOC_Os04g50920*. Colour bar indicates the scaled expression level

While these analyses allowed the reconstruction of potential regulatory modules active in each cell type of the rice root, they were insufficient to reveal developmental dependencies between TF and target cell types. Pseudotime analysis has been a major step towards establishing causal relations in scRNA-seq data, and we have previously used it to reveal the differentiation trajectory of root hairs from epidermal cells [7]. Importantly, these analyses can also be applied to scATAC-seq data to order ACR heterogeneity within a cell type cluster into time-resolved chromatin accessibility dynamics. Using the root hair cluster as a model, we identified 13,848 ACRs, 131 TF motifs, and 3882 genes with significant differences in chromatin accessibility across the root hair pseudotime trajectory (Additional file 1: Fig. S7). Several known root hair developmental genes, including *LOC_Os10g42750*, were identified among the top differentially accessible genes throughout root hair development. Consistent with the central role of this gene for root hair development [23], the accessibility of chromatin in its regulatory region strongly increased during the transition from the epidermis to root hair identity, which was consistent with its RNA expression profile derived from scRNA-seq (Fig. 3a, b) [7]. Interestingly, we found a WRKY-binding motif in the ACR of *LOC_Os10g42750*, and cross-referencing with our scRNA-seq data allowed us to identify the WRKY TF *LOC_Os04g50920*, which is only expressed in the root hair and root cap and thus may represent a potential upstream activator (Fig. 3c, d). Thus, the pseudotime analysis of chromatin accessibility and RNA expression not only was able to resolve gene expression dynamics during root hair development but also allowed to resolve the regulatory mechanisms underlying a key developmental transition.

### Single-cell chromatin accessibility dynamics in response to heat stress

**Fig. 4:**
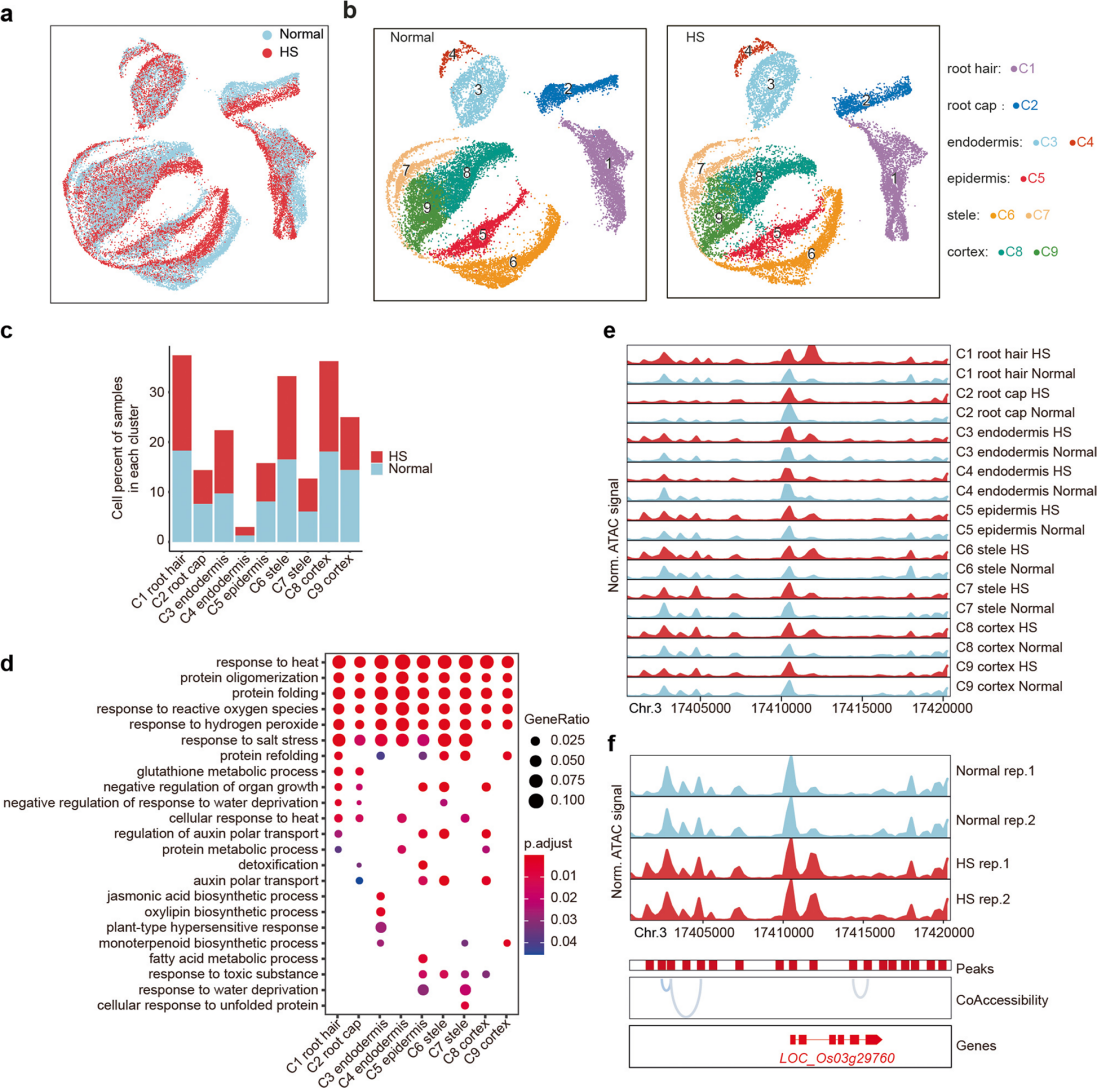
Single-cell chromatin accessibility dynamics in response to heat stress. **a** UMAP visualization of normal and HS scATAC clusters after alignment. Each dot indicates a single cell. The colours indicate normal or HS. **b** UMAP visualization of separated normal and HS samples of scATAC-seq clusters after alignment. Each dot indicates a single cell. **c** Bar plot comparison of cell percentage between normal and HS for each cluster. **d** Scatter plots of GO enrichment analysis of differentially accessible genes for each cluster between normal and HS. Overrepresentation analysis and visualization performed using the ClusterProfiler R package. **e**, **f** Genome browser screenshot comparison of chromatin accessibility between normal and HS samples from bulk ATAC-seq (**f**) and scATAC-seq with each cluster (**e**)

An important aspect of plant development is its astounding plasticity in response to environmental variation. Therefore, we were interested to analyse how an environmental factor, such as temperature, would affect the chromatin accessibility and hence the transcriptional programmes leading to root cell type development. We have previously shown that chromatin accessibility is responsive to heat stress (HS) in rice roots but were lacking cell type resolution [24]. To explore the HS-induced chromatin dynamics at the single-cell level, we carried out two independent rounds of scATAC-seq on rice root tips after exposure of 3 h to 45 °C (Fig. 4, Additional file 1: Fig. S2; Additional file 2: Tables S8, S9). In total, we generated scATAC-seq profiles from 21,446 cells, which yielded on average 16,570 unique fragments mapping to the Nip genome (Additional file 1: Table S1). For comparative analysis of cells that had not experienced heat shock, scATAC-seq reads from control and HS samples were merged and subsequently clustered. UMAP visualization revealed nine clusters of HS-treated cells that overall were similar to clusters identified in controls (Fig. 4a, b). In addition to the general alignment of cell clusters, the relative numbers of cells per cluster were also comparable (Fig. 4c), demonstrating that chromatin accessibility was not massively disturbed by HS. Interestingly, we observed that cells of the root cap, root hair, and stele showed more divergence between normal growth conditions and HS (Fig. 4e) than those of the epidermis, endodermis, and cortex, suggesting chromatin accessibility in these tissues was specifically responsive to heat stress. To elucidate the mechanisms driving specific and more general responses to temperature, we identified heat shock-specific ACRs and searched for overrepresented regulatory motifs by MotifScan [25]. Notably, heat stress transcription factor (HSF)-binding motifs were the top enriched ones for all nine cell types (Additional file 3: Table S10). In the next step, we subjected genes associated with heat shock-specific ACRs to GO analysis and found that several GO terms were shared by all cell clusters (Fig. 4d). Reassuringly, “response to heat” was the top GO term across all cell types, demonstrating that HS generally affected the chromatin accessibility of heat response-related genes. Interestingly, we also observed cluster-specific GO terms. For example, “jasmonic acid biosynthetic process” was found in cluster 3 endodermis (Fig. 4d; Additional file 1: Fig. S8). To compare our scATAC and bulk ATAC data, we used gene track analysis on genes that showed responses in chromatin state in one of the assays (Fig. 4e, f), while we were able to identify many differential ATAC peaks between normal and HS that were cell type-specific in scATAC-seq (Fig. 4e), which could not be identified by bulk ATAC-seq (Fig. 4f), suggesting scATAC-seq not only offers single-cell resolution, but also is more powerful in identifying ACRs in response to environmental changes.

## Conclusions

In this study, we applied scATAC-seq and obtained chromatin profiles of more than 46,000 nuclei of rice root tips under normal and heat stress conditions. Single-cell chromatin landscapes allowed us to identify nine clusters of cells that covered the major cell types found in the root tip. This structure correlated well with scRNA-seq data and revealed cell type-specific regulatory modules. Importantly, our analysis also defined chromatin dynamics during cell type development and in response to heat stress. Taken together, our results represent a valuable resource of chromatin accessibility at single-cell resolution to study the development and function of rice root cell types.

## Methods

### Rice growth conditions and nuclei isolation

Rice Nip seeds were soaked in water in the dark until germination and then cultured in Hoagland’s Complete Nutrient Solution for 3 days (28 °C, 10-h light/14-h dark cycles, 300 lux). Primary root tips 5 mm in length from the 3-day-old rice seedlings were harvested and fixed with cold paraformaldehyde solution (2%) for 30 min. Then, the root tips were transferred to a Dounce grinder (kimble), which was filled with cold lysis buffer (10 mM Tris-HCl, 10 mM NaCl, 1 mM MgCl_2_, 1% BSA) and 0.2% Tween 20 (0.2% NP-40, 0.0075% digitonin). 0.5% 2-ME (v/v) were added, and then the roots were ground completely. The homogenate was filtered with a 20-μm mesh (Sysmex) following centrifuge at 480 rcf for 5 min at 4 °C. After removing the supernatant, 1% BSA buffer (10 mM Tris-HCl (pH7.4), 10 mM NaCl, 3 mM MgCl_2_, 1% BSA (v/v)) was added to resuspend the pellet. Then, the mixture was placed on 80% Percoll and following centrifuge at 300 rcf for 25 min at 4 °C. After removing 150 μl supernatant, the nuclei were transferred to a new Eppendorf tube and washed twice with 1% BSA buffer. The nuclei were resuspended in a chilled Diluted Nuclei Buffer (10x Genomics, PN-2000153). The nuclei were stained by DAPI and trypan to check the quality and quantity.

### scATAC-seq library preparation and sequencing

The isolated nuclei were processed immediately through the 10x Chromium Single Cell Platform according to the manufacturer’s instructions (10x Genomics). Briefly, 16,000 resuspended nuclei (concentration 3000–7000 nuclei/μl) were combined with ATAC Buffer and ATAC Tn5 transposase (10x Genomics) to form a transposition mix. After incubation for 30 min at 37 °C, barcoding reagent and enzyme (10x Genomics) were added, and the resulting mix was loaded onto a single-cell chip, which was subsequently placed into a Chromium Single Cell Controller instrument for library construction. Quantification of the DNA library was done by Agilent 2100 Bioanalyzer then sequenced with an Illumina Hiseq 2000 sequencer.

### Bulk ATAC-seq and data processing

Bulk ATAC-seq was performed as described previously [24]. Qubit fluorometer and Agilent Bioanalyzer 2100 were used to check library quality and concentration. DNA libraries were constructed using NEBNext DNA Library Prep Kit (Neb) and sequenced on an Illumina HiSeq 2000 platform with 150-bp paired-end reads. FASTQ files were mapped to *Oryza sativa* Nip genome by using Bowtie2 [26] with “-N 1 -X 2000” parameters. The MACS2 software was used to call peaks with -q 0.05 --nomodel --extsize 150. We defined differential peaks between two groups with the MAnorm software [27] by using abs (*M* value) ≥ 1; *M* value is the log2 fold change of normalization chromatin accessibility signal between two normal and HS samples.

### Single-cell ATAC-seq data processing (QC)

FASTQ files of each sample were firstly processed by using cellranger-atac (v.1.2.0) count pipeline coupled with *Oryza sativa* Nip genome to generate fragment and cell files. Next, we used createArrowFiles function in ArchR [19] to filter low-quality cells following these criteria: (1) filterTSS = 1.5; (2) filterFrags = 1000; and (3) validBarcodes = cellranger.cells. The cellranger.cells was generated with getValidBarcodes function in ArchR, which means we only used the cells after passing the strict quality control with cellranger-atac and ArchR softwares. At last, doublets (5174 cells) were filtered by addDoubletScores and filterDoublets functions with default parameters in ArchR. We used the ArchR software for most of our single-cell ATAC-seq datasets analysis.

### Estimation of gene accessibility scores

In view of smaller genome of rice than human genomes, we estimated the gene accessibility score by using addGeneScoreMatrix function with “exp(-abs(x)/2000) + exp(-1)” geneModel, “c(500, 10000)” extendstreams and “2000” geneUpstream parameters. ArchR allows for the use of accessibility within the entire gene body and putative distal regulatory elements with complex user-supplied custom distance-weighted accessibility models contributes to the gene score.

### Clustering and cell type annotation

We got the “TileMatrix”, which is genome-wide 500-bp tiles in ArchRProject. log(TF-IDF) (Term frequency-inverse document frequency) normalization with TileMatrix, Latent Semantic Indexing (LSI) dimensionality reduction and harmony batch effect correction methods were chosen for the subsequent analysis. We used 0.1 resolution to define cell clusters and UMAP to visualize single cells. Nine clusters were identified and annotated cell types based on the promoter of cell type marker genes containing cluster-specific ACRs.

### ACRs and differential chromatin accessibility identification

MACS2 was used to call ATAC peaks. Specifically, we generated a reproducible peak set and called union peaks among clusters with addReproduciblePeakSet and addPeakMatrix functions, respectively. The getMarkerFeatures function was used to detect all kinds of differential chromatin accessibility in our analysis, in more detail, GeneScoreMatrix for defining cluster-specific marker genes and PeakMatrix for detecting cluster-specific ACRs among clusters. On the other hand, differential peaks and gene scores were also evaluated between the normal and HS groups in each cluster. Bias with TSS enrichment and log10(nFrags) were also accounted for in selecting a matched null group for marker feature identification, and the Wilcoxon test was used for statistical analysis.

### RNA in situ hybridization

RNA in situ hybridization was performed as described previously [28, 29]. Briefly, the specific region for the selected genes was cloned into pGEM-TEasy (Promega) vector, and then Digoxigenin RNA labelling kit (Roche) was used for in vitro transcription and labelling. After hybridization and immunological detection, signals were visualized under Leica DM6 B microscopy with bright-field mode. Primers of 5′-GCTGATATCCTTGCTCTGGTAGC-3′ and 5′-TAATACGACTCACTATAGGGACCTGATCCTTTGCGTCAAGG-3′ were used to generate an antisense probe of *LOC_Os04g53640*, and primers 5′-TAATACGACTCACTATAGGGGCTGATATCCTTGCTCTGGTAGC-3′ and 5′-ACCTGATCCTTTGCGTCAAGG-3′ were used to generate its sense probe.

### Functional enrichment

We used cutoff (FDR ≤ 0.01 and Log2FC ≥ 1) to define cluster-specific marker genes and peaks, and the nearest gene of a peak was regarded as its target gene for functional analysis. compareCluster function in the clusterProfiler package [30, 31] was used to do functional analysis with differential gene sets annotated with GO database (Gene Ontology, http://geneontology.org/). Enrichment terms were obtained with p.adjust < 0.05. *p*-value adjustment (FDR) is performed using the Benjamini–Hochberg method. Here, enrichment is implemented with the hypergeometric test (Fisher’s exact test).

### Identification of co-accessible ACRs

We defined co-accessibility ACRs with a correlation of 0.5 threshold using addCoAccessibility and getCoAccessibility functions in ArchR across all cells. Co-accessibility ACRs will be shown as a loop when plotting a browser tracks with ArchRProject.

### Motif enrichment analysis and deviation score assessment

Rice TF motifs were downloaded from the PlantTFDB v5.0 database (http://planttfdb.gao-lab.org/download.php#bind_motif). First, we used MotifScan (http://bioinfo.sibs.ac.cn/shaolab/motifscan/index.php) to predict all TF motif-binding sites across union peak regions with cutoff *p*-value < 1e−4. “genomecompile” and “motifcompile” commands had been run to get reference index and background motif score cutoff under different *p*-value. Next, we used the addPeakAnnotations function to add our motif-binding site annotation and got a motif deviation score matrix with the addDeviationsMatrix function by ArchR.

### Trajectory analysis and changes of features across pseudotime

We performed trajectory analysis by using the addTrajectory function in the ArchR software [19] with default parameters. The root hair cells are differentiated from the epidermis; thus, we defined a trajectory backbone (C5>C1, as the addTrajectory function in ArchR must span at least 3 groups, we subcluster cells with resolution 0.3, which C1 was exactly divided into two parts) that provides a rough ordering of cell clusters. Changes in the features about gene score, motif, and peak across pseudotime were calculated by using the getTrajectory function with default suggested parameters of ArchR official tutorial (https://www.archrproject.com/bookdown). The trajectory-associated figures were plotted by using plotTrajectoryHeatmap and plotTrajectory functions. Some cells were filtered in the final trajectory by preFilterQuantile with the default parameters, which resulted in these cells were not in the final trajectory.

### Integration of scRNA-seq and scATAC-seq datasets

We integrated our scRNA-seq [7] with scATAC-seq data by adopting the approach used for integrating *Arabidopsis* scRNA-seq and scATAC-seq datasets [16]. Firstly, we created a seurat object by using a gene-score matrix from scATAC-seq data, and the CCA method was chosen for the integration by using FindTransferAnchors to get a transfer.anchors object. Secondly, the TransferData function was used to add the imputed data matrix to the scATAC-seq object in LSI reduction weight, and variable genes of scRNA-seq object were used. Finally, the scRNA-seq and scATAC-seq object were merged, and we performed UMAP on this combined object to visualize the co-embedding.

### Integration of ACRs and histone modification datasets

The ChIP-seq datasets, including H3K4me1, H3K4me3, H3K27me3, and H3K9me2 from 1-week-old rice Nip, were downloaded from http://glab.hzau.edu.cn/RiceENCODE/ [32, 33]. Python package deeptools was used to generate the plot.

## Supplementary Information

**Additional file 1: Figure S1.** Isolation and purification of nuclei from rice root tips. **Figure S2.** Evaluation and quality control of rice scATAC-seq. **Figure S3.** Example of cluster-enriched chromatin accessibility and surrounding genes, and in situ hybridization of sense probe. **Figure S4.** Correlation between scATAC-seq and scRNA-seq. **Figure S5.** Heatmap showing enrichment of TF motifs. **Figure S6.** TFs and motif enrichment at ACRs in rice root. **Figure S7.** Pseudotime heatmap ordering from epidermis to root hair. **Figure S8.** Violin plot showing chromatin accessibility changes in genes related to “jasmonic acid biosynthetic process”. **Table S1:** Sequencing statistics.

**Additional file 2: Table S2:** Marker genes of each cluster. **Table S3:** ACRs of each cluster for Normal and HS. **Table S4:** List of genes with open chromatin accessibility but low/no expression. **Table S5:** List of genes with close chromatin accessibility but high expression. **Table S6:** List of overlapped marker genes between scRNA-seq and scATAC-seq. **Table S7:** List of GO terms for genes associated with cluster specific ACRs. **Table S8:** ACRs and corresponding genes detected in the bulk ATAC-seq experiment for Normal. **Table S9:** ACRs and corresponding genes detected in the bulk ATAC-seq experiment for HS.

**Additional file 3: Table S10:** Enriched TF motifs in differential ACRs upon heat stress for each cluster. (XLS 403 kb)

## Data Availability

All data generated or analysed during this study are included in this published article, its supplementary information files, and publicly available repositories. The scATAC-seq and bulk ATAC-seq data in this study have been deposited in the NCBI Gene Expression Omnibus (GEO) database with the accession numbers: GSE214132.

## Abbreviations

ACRs: Accessible chromatin regions
ATAC-seq: Assay for transposase-accessible chromatin with high-throughput sequencing
Dof: DNA-binding with one finger
GEO: Gene expression omnibus
GO: Gene Ontology
HS: Heat stress
HSFs: Heat stress transcription factors
LSI: Latent semantic indexing
Nip: Nipponbare
QC: Quality control
SCAR: Single-cell chromatin accessibility of rice root
scATAC-seq: Single-cell ATAC-seq
scRNA-seq: Single-cell RNA sequencing
TF-IDF: Term frequency-inverse document frequency
TFs: Transcription factors
TSS: Transcription start site
UMAP: Uniform manifold approximation and projection
