# Supplementary material for: Chromatin accessibility illuminates single-cell regulatory dynamics of rice root tips
Source: BMC Biol. 2022 Dec 8;20:274. doi: 10.1186/s12915-022-01473-2 (PMC9733338; doi:10.1186/s12915-022-01473-2)
Supplement: Supplementary file 1 — Additional file 1: Figure S1. Isolation and purification of nuclei from rice root tips. Figure S2. Evaluation and quality control of rice scATAC-seq. Figure S3. Example of cluster-enriched chromatin accessibility and surrounding genes, and in situ hybridization of sense probe. Figure S4. Correlation between scATAC-seq and scRNA-seq. Figure S5. Heatmap showing enrichment of TF motifs. Figure S6. TFs and motif enrichment at ACRs in rice root. Figure S7. Pseudotime heatmap ordering from epidermis to root hair. Figure S8. Violin plot showing chromatin accessibility changes in genes related to “jasmonic acid biosynthetic process”. Table S1: Sequencing statistics. [file 12915_2022_1473_MOESM1_ESM.docx]

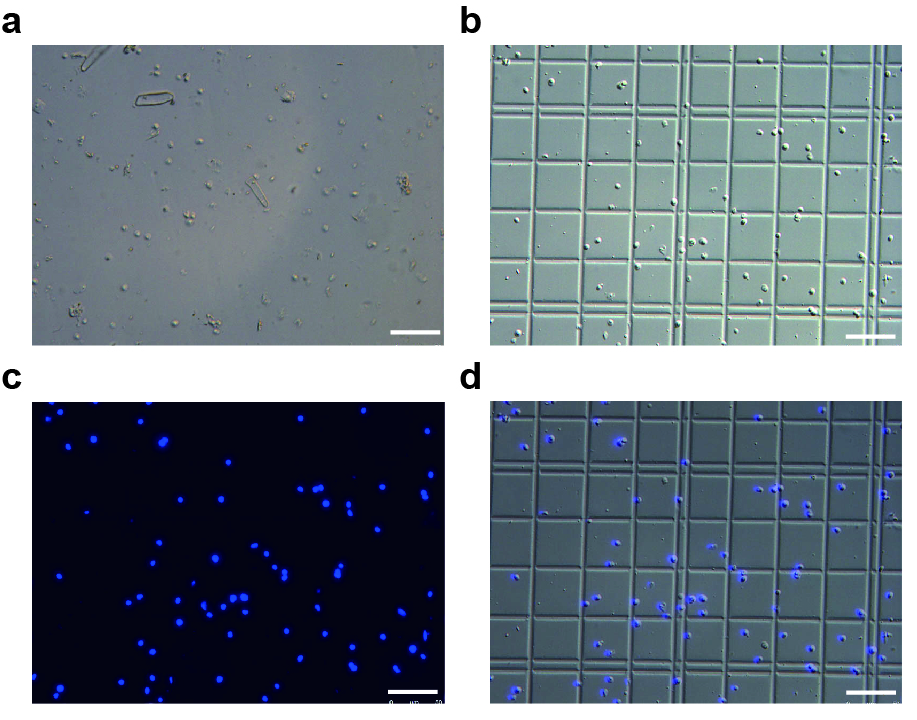


**Figure S1.** Isolation and purification of nuclei from rice root tips. **a** Before nuclei purification, nuclei were mixed with tissue fragments, debris of membrane and cell wall, and starch grain in the lysate. **b-d** Nuclei with high quality and purification. The nuclei were visualized under DIC mode of Leica DM6 B microscope. Tissue fragments, debris and starch grain have been removed (**b**). Intact nucleus showed intensive blue light, indicating the nuclei (**c** dark field and **d** merged) were almost intergral. DAPI was used for nuclei staining. Scale bars, 50 μm.


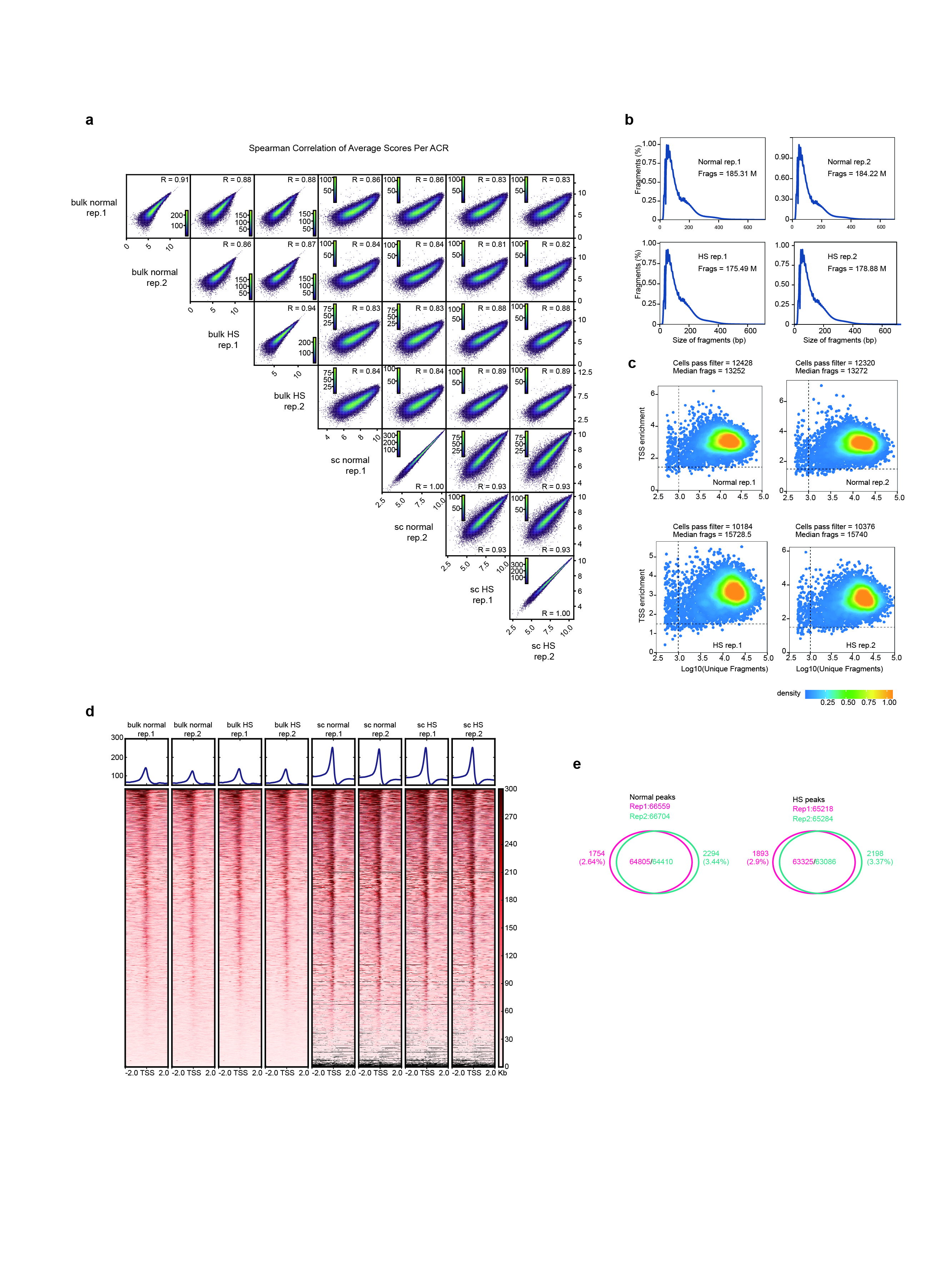


**Figure S2.** Evaluation and quality control of rice scATAC-seq. **a** Scatter plots of pairwise comparisons of accessibility (median correlation for each class shown) for all the ATAC-seq samples in this study. Log10 reads per million (RPM) unique mapped reads in the union of all tissue-level peaks shown. The value of correlation coefficient (R) was indicated. Colour bars indicate number of of ACRs. **b** Distribution of sequenced fragment length for each single cell ATAC-seq library. **c** Scatter plots showing the number of unique ATAC-seq fragments in each single cell (each dot) compared with TSS enrichment of all fragments in that cell. Dashed lines show the cutoff for high-quality single-cell data (1,000 unique fragments and TSS score greater than or equal to 1.5). Density is given in arbitrary units. **d** Enrichment plots centered on TSSs in 200 bp windows for each ATAC-seq sample. **e** Venn diagram showing the overlapping peaks between scATAC-seq replicates.


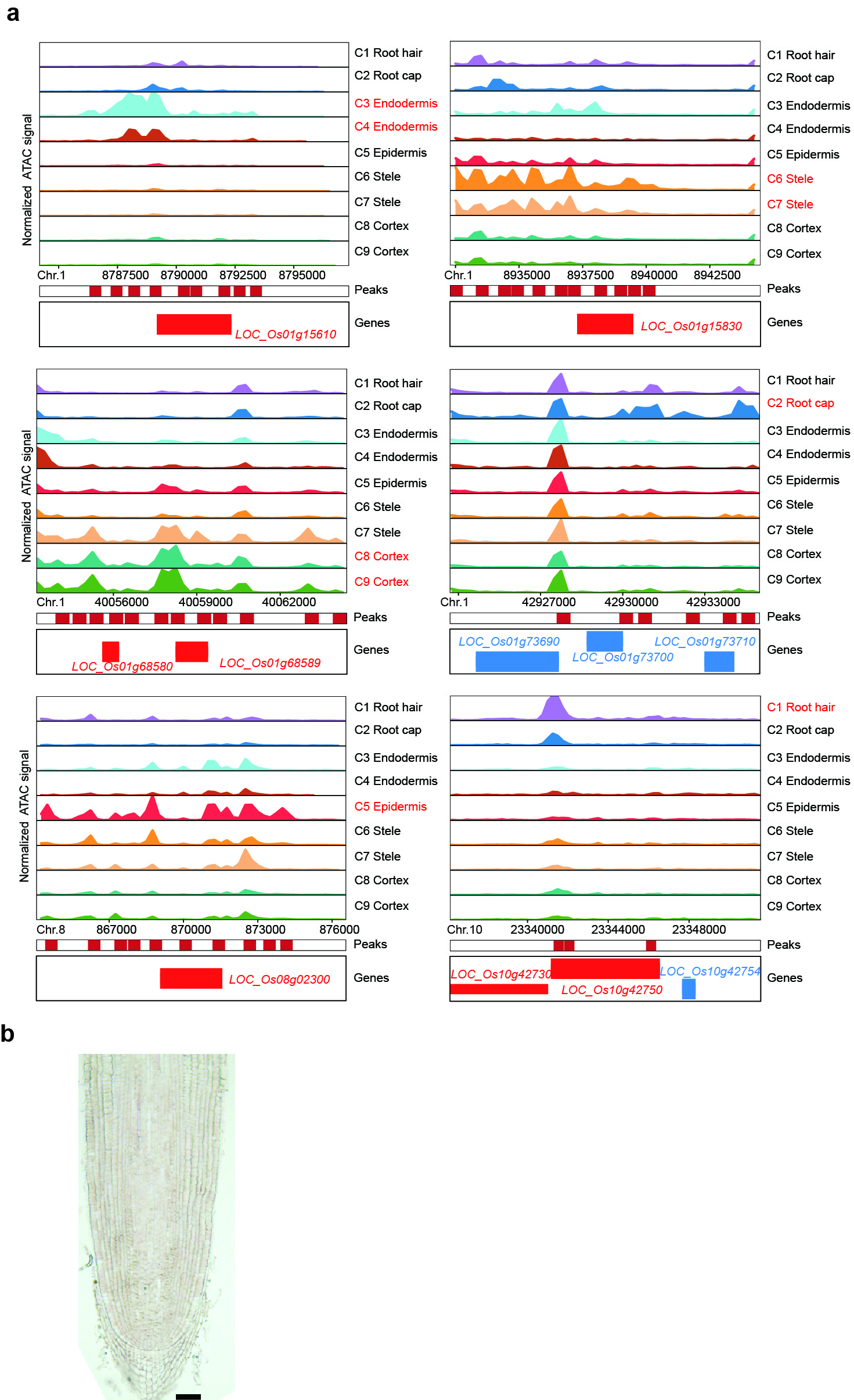


**Figure S3.** Example of cluster-enriched chromatin accessibility and surrounding genes, and in situ hybridization of sense probe. **a** Genome tracks of representative cluster-enriched chromatin accessibility and surrounding genes for each cluster. The enriched cluster were highlighted. **b** RNA in situ hybridization of *LOC_Os04g53640* using sense probe. Scale bar, 40 μm.

**
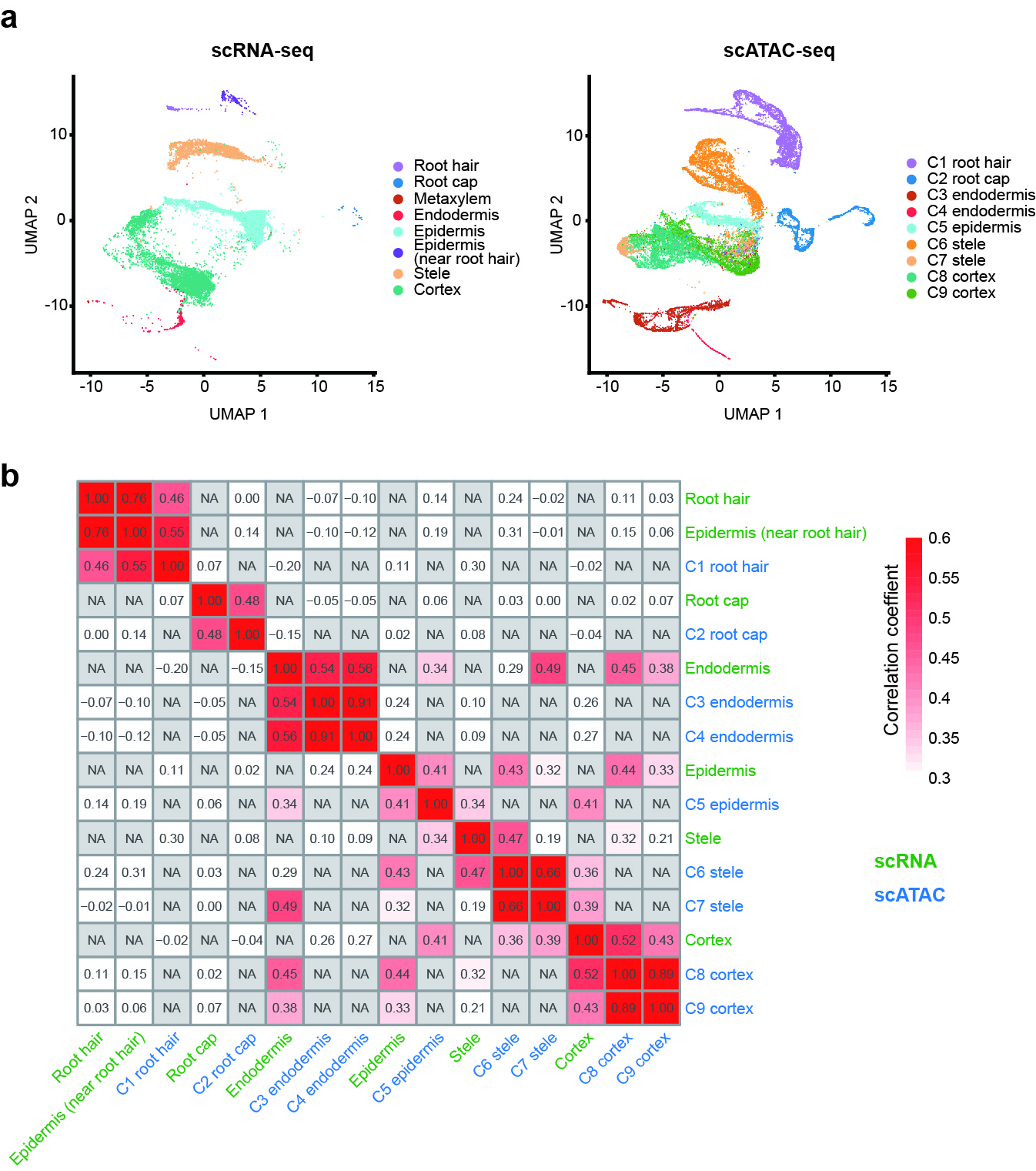
**

**Figure S4.** Correlation between scATAC-seq and scRNA-seq. **a** After co-embedding of root tip scATAC-seq cells and scRNA-seq datasets, UMAP showing scRNA-seq cells (left) and scATAC-seq cells (right), respectively. **b** Heatmap showing correlation coefficient of marker genes for each cell type between scATAC-seq and scRNA-seq.


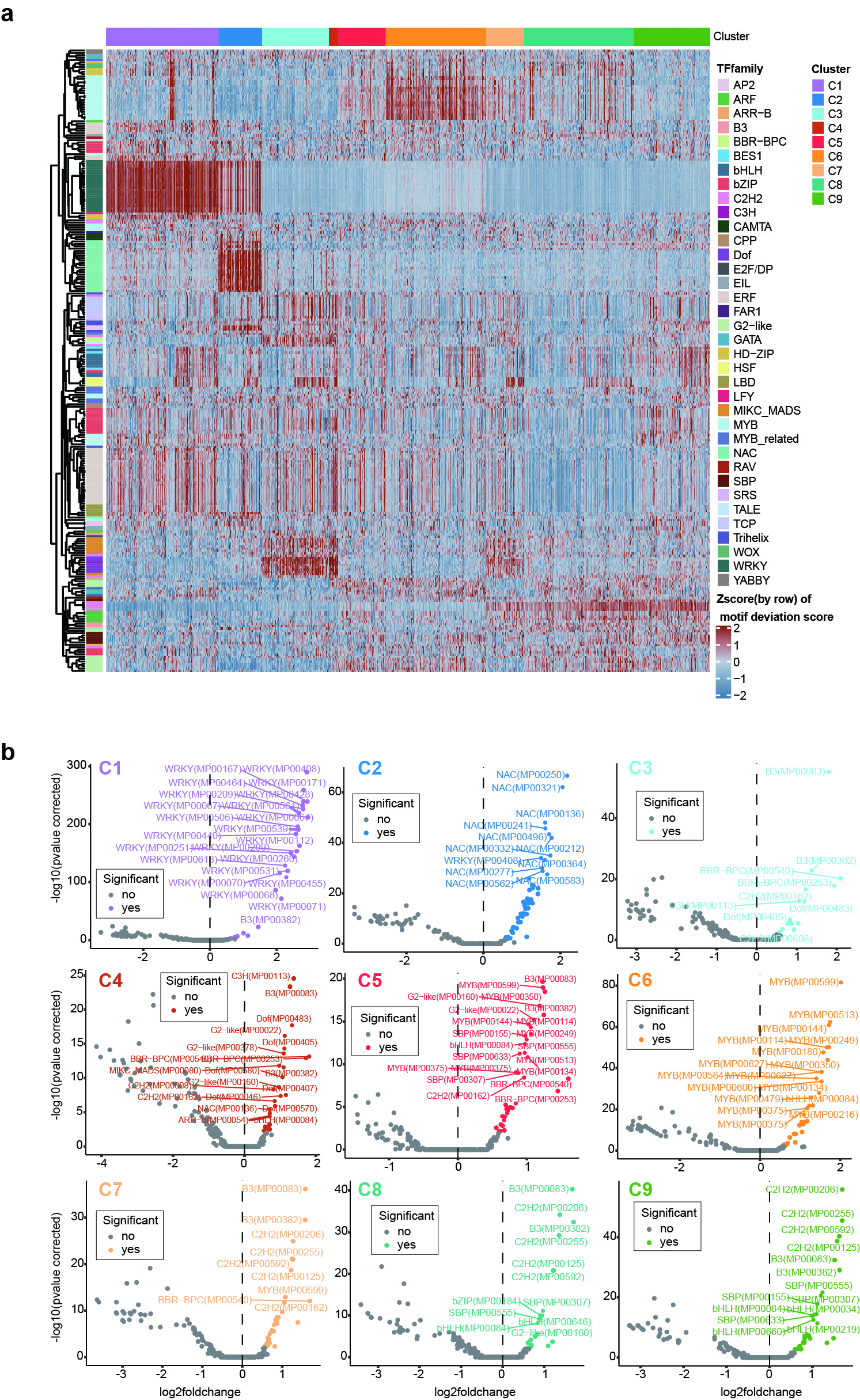


**Figure S5.** Heatmap showing enrichment of TF motifs. **a** TF motif deviations for 264 TF motifs (rows) per nucleus (columns, 38,349 cells). The TF family for each corresponding motif is denoted by row header colors on the right side of the heatmap. Cluster identities are illustrated as column header colors on the top. **b** Scatter plot of motif enrichment showing top 2,000 specific ACRs for each cell type cluster. Significant (corrected p value < 0.01) motifs were colored. P values were calculated by using Benjamini–Hochberg method after fisher-exact test with random regions.


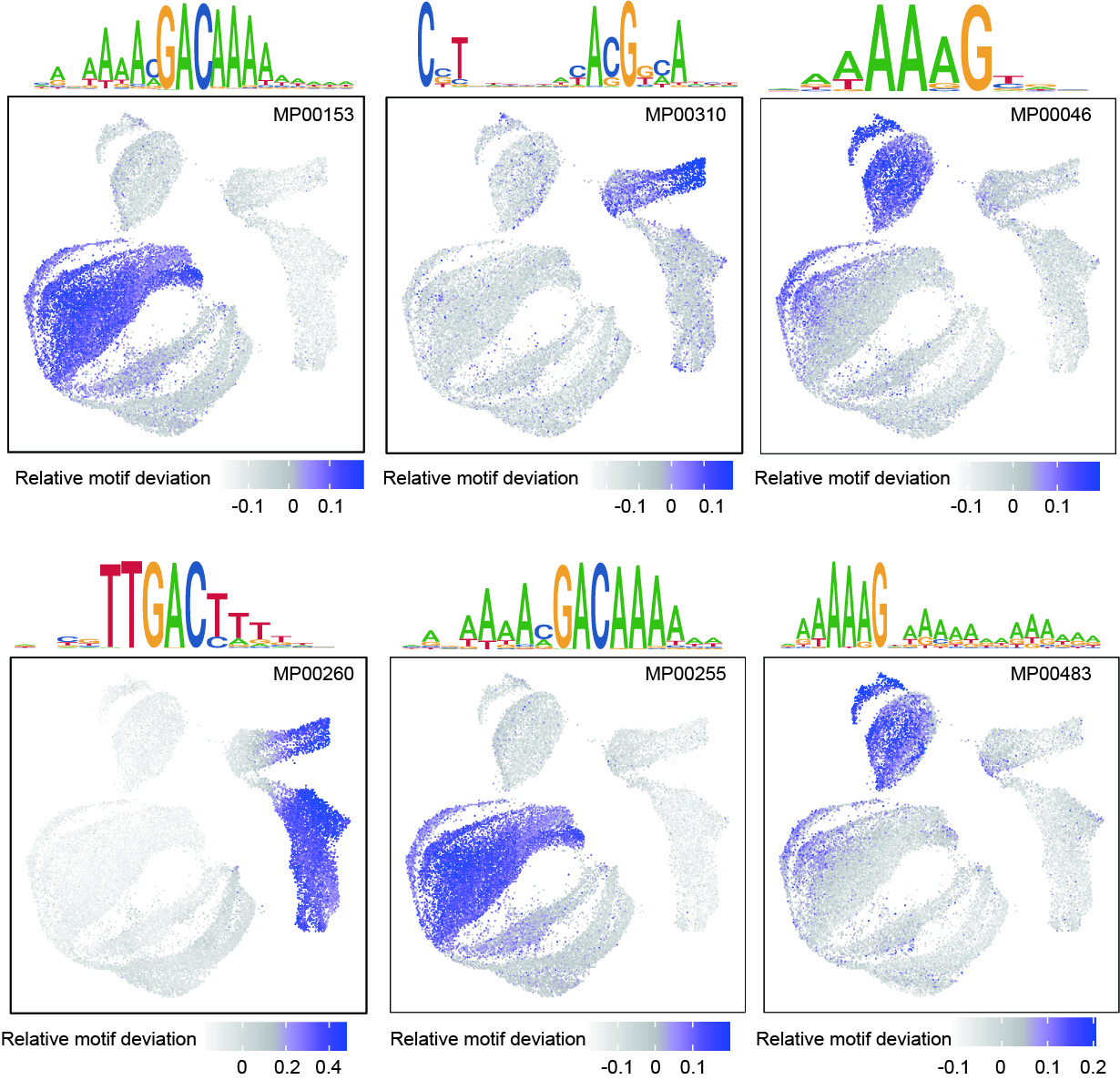


**Figure S6.** TFs and motif enrichment at ACRs in rice root. Examples of UMAP visualization of gene accessibility for TFs and their associated motif deviations.

**
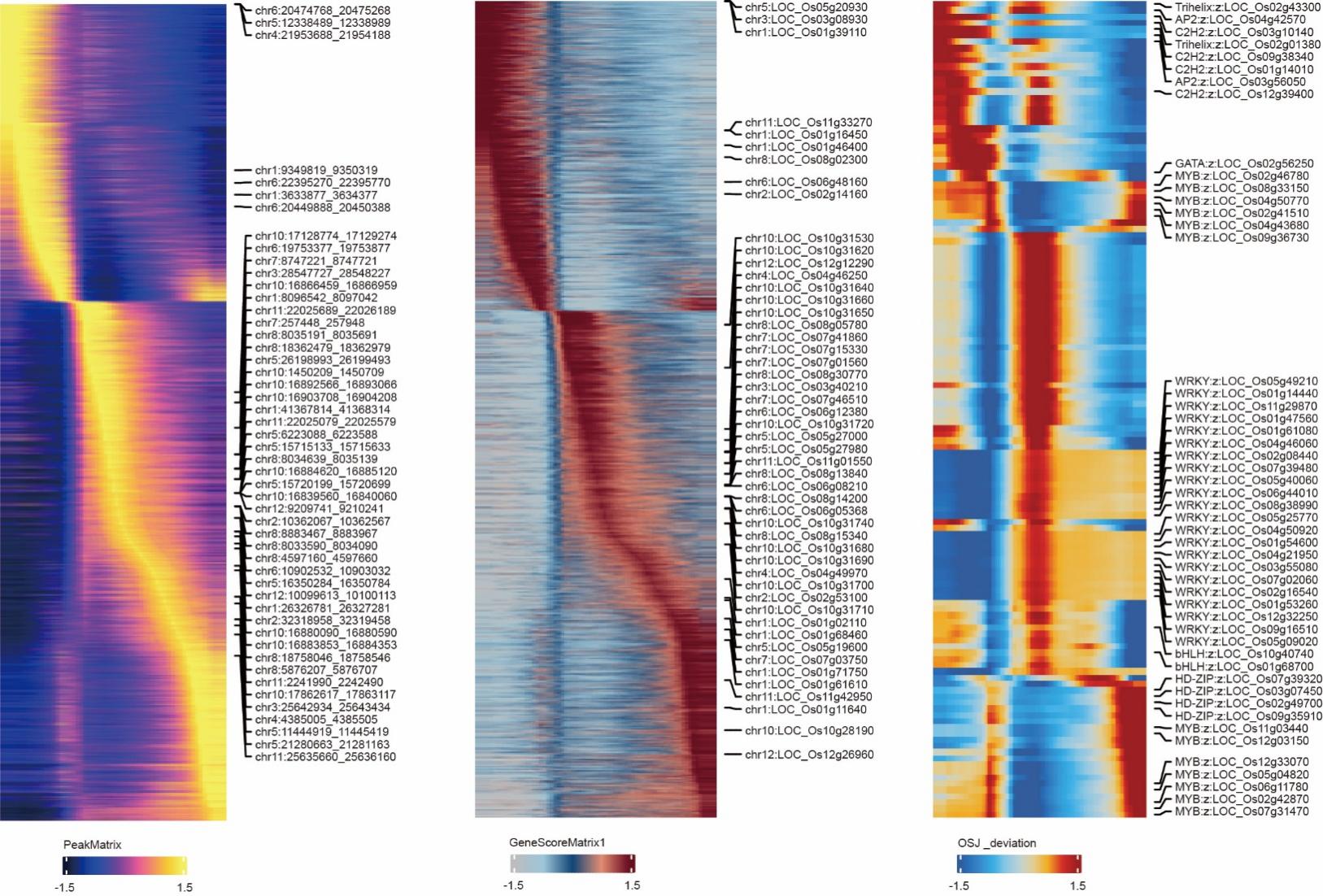
**

**Figure S7.** Pseudotime heatmap ordering from epidermis (left) to root hair (right). Relative motif deviations for 13,848 ACRs (left), gene accessibility for 3,882 TFs (middle), and accessibility of 131 TF motifs (right) associated with pseudotime (x axis).

**
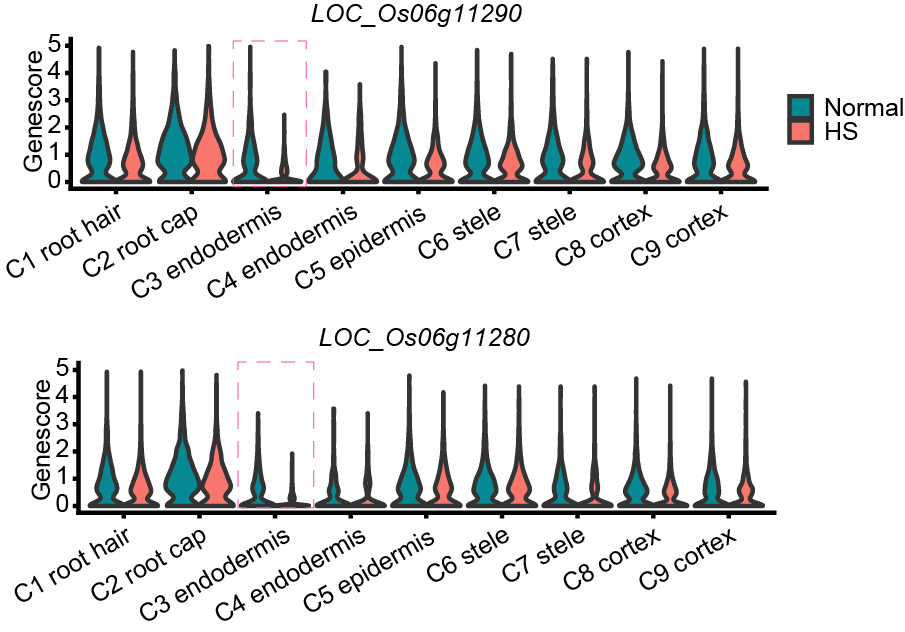
**

**Figure S8.** Violin plot showing chromatin accessibility changes in genes related to “jasmonic acid biosynthetic process”. Most significant changes were observed in C3 endodermis.

**Table S1. Sequencing statistics.**

| **Sample** | **Total PE reads** | **Estimated number of cells** | **Total usable fragments (Unique Tn5 )** | **Fraction of fragments overlapping TSS** | **Mean fragments per cell** |
| --- | --- | --- | --- | --- | --- |
| Normal rep.1 | 453,133,782 | 12,708 | 185,346,742 | 55.36% | 14,585 |
| Normal rep.2 | 458,094,333 | 12,604 | 184,262,285 | 55.38% | 14,619 |
| HS rep.1 | 477,886,588 | 10,719 | 176,043,343 | 55.48% | 16,423 |
| HS rep.2 | 472,183,593 | 10,727 | 179,307,581 | 55.45% | 16,715 |
